# Supplementary material for: Structural basis for allosteric control of the SERCA-Phospholamban membrane complex by Ca2+ and phosphorylation
Source: eLife. 2021 May 12;10:e66226. doi: 10.7554/eLife.66226 (PMC8184213; doi:10.7554/eLife.66226)
Supplement: Transparent reporting form [file elife-66226-transrepform.docx]

***eLife’s* transparent reporting form**

We encourage authors to provide detailed information *within their submission* to facilitate the interpretation and replication of experiments. Authors can upload supporting documentation to indicate the use of appropriate reporting guidelines for health-related research (see [EQUATOR Network](http://www.equator-network.org/%20)), life science research (see the [BioSharing Information Resource](https://biosharing.org/)), or the [ARRIVE guidelines](http://www.plosbiology.org/article/info:doi/10.1371/journal.pbio.1000412) for reporting work involving animal research. Where applicable, authors should refer to any relevant reporting standards documents in this form.

If you have any questions, please consult our Journal Policies and/or contact us: [editorial@elifesciences.org](mailto:editorial@elifesciences.org).

**Sample-size estimation**

- You should state whether an appropriate sample size was computed when the study was being designed
- You should state the statistical method of sample size computation and any required assumptions
- If no explicit power analysis was used, you should describe how you decided what sample (replicate) size (number) to use

Please outline where this information can be found within the submission (e.g., sections or figure legends), or explain why this information doesn’t apply to your submission:

Sample sizes were not applicable for structural data obtained from NMR experiments, which were conducted with enough scans to achieve signal/noise ratios >= 3. Dynamic refinement calculations were done at timescales long enough to converge upon experimental restraints.

**Replicates**

- You should report how often each experiment was performed
- You should include a definition of biological versus technical replication
- The data obtained should be provided and sufficient information should be provided to indicate the number of independent biological and/or technical replicates
- If you encountered any outliers, you should describe how these were handled
- Criteria for exclusion/inclusion of data should be clearly stated
- High-throughput sequence data should be uploaded before submission, with a private link for reviewers provided (these are available from both GEO and ArrayExpress)

Please outline where this information can be found within the submission (e.g., sections or figure legends), or explain why this information doesn’t apply to your submission:

Technical replicates of NMR spectra of PLN in bicelles were implicit with selectively labelled samples (4 for non-phosphorylated and 3 for phosphorylated; see Supp. Figure 2). These replicates were vital for confirming the existence and nature of the excited topological state. Structural ensembles were computed by a replica-averaged (8 replicas) approach described in the Methods. Biological replicates do not apply to this work since no cell- or organism-based experiments are reported.

**Statistical reporting**

- Statistical analysis methods should be described and justified
- Raw data should be presented in figures whenever informative to do so (typically when N per group is less than 10)
- For each experiment, you should identify the statistical tests used, exact values of N, definitions of center, methods of multiple test correction, and dispersion and precision measures (e.g., mean, median, SD, SEM, confidence intervals; and, for the major substantive results, a measure of effect size (e.g., Pearson's r, Cohen's d)
- Report exact p-values wherever possible alongside the summary statistics and 95% confidence intervals. These should be reported for all key questions and not only when the p-value is less than 0.05.

Please outline where this information can be found within the submission (e.g., sections or figure legends), or explain why this information doesn’t apply to your submission:

P values (determined using unpaired T-tests) for all comparisons between two datasets made within the main text are quoted in parenthesis. This applies to comparing PLN topology (tilt and azimuthal angles) between non-phosphorylated, phosphorylated and SERCA-bound forms, in which the error of each topological fit are estimated based on a bootstrapping analysis described in the Supplementary Methods. P-values were also reported for activity assays in Figure S12 and described in the caption.

(For large datasets, or papers with a very large number of statistical tests, you may upload a single table file with tests, Ns, etc., with reference to sections in the manuscript.)

**Group allocation**

- Indicate how samples were allocated into experimental groups (in the case of clinical studies, please specify allocation to treatment method); if randomization was used, please also state if restricted randomization was applied
- Indicate if masking was used during group allocation, data collection and/or data analysis

Please outline where this information can be found within the submission (e.g., sections or figure legends), or explain why this information doesn’t apply to your submission:

Not relevant as this study does not contain clinical data.

**Additional data files (“source data”)**

- We encourage you to upload relevant additional data files, such as numerical data that are represented as a graph in a figure, or as a summary table
- Where provided, these should be in the most useful format, and they can be uploaded as “Source data” files linked to a main figure or table
- Include model definition files including the full list of parameters used
- Include code used for data analysis (e.g., R, MatLab)
- Avoid stating that data files are “available upon request”

Please indicate the figures or tables for which source data files have been provided:

* All experimental NMR and modelling data related to this work are currently being made publicly from the Data Repository for University of Minnesota (DRUM).

* NMR chemical shift and dipolar couplings for samples (PLN, pPLN, PLN + SERCA and pPLN + SERCA) are currently being deposited on the Biological Magnetic Resonance Data Bank (BMRB). The deposition will include raw spectral data and processing parameters. These data will also be made available on DRUM.

* Table 1 in the Supplementary material summarizes chemical shifts and dipolar couplings collected from this work.

* Representative PLN/SERCA structures from each cluster identified in ROAR-MD refined ensemble are to be deposited on the Protein Data Bank. These structures will also be available on DRUM.
